# Deconstructing the eradication of new world screwworm in North America: retrospective analysis and climate warming effects

**DOI:** 10.1111/mve.12362

**Published:** 2019-02-13

**Authors:** A. P. Gutierrez, L. Ponti, P. A. Arias

**Affiliations:** ^1^ Center for the Analysis of Sustainable Agricultural Systems (CASAS Global) Kensington CA U.S.A.; ^2^ Division of Ecosystem Science, College of Natural Resources University of California Berkeley CA U.S.A.; ^3^ Agenzia nazionale per le nuove tecnologie, l'energia e lo sviluppo economico sostenibile (ENEA), Centro Ricerche Casaccia Rome Italy; ^4^ Grupo de Ingeniería y Gestión Ambiental (GIGA), Escuela Ambiental, Facultad de Ingeniería, Universidad de Antioquia Medellín Colombia

**Keywords:** Allee effect, climate change, eradication, physiologically based modelling, sterile insect technique

## Abstract

Before its eradication from North America, the subtropical‐tropical new world screwworm fly *Cochliomyia hominivorax* (Coquerel) invaded southwestern temperate areas of the U.S.A., where it caused myiasis in wildlife and livestock. Outbreaks of the fly occurred during years when adult migrants were carried northward on North American monsoon winds from the northern areas of Mexico and south Texas. We deconstruct, retrospectively, the biology and the effect of weather on the eradication of the fly in North America. Screwworm was found to be an ideal candidate for eradication using the sterile insect technique (SIT) because females mate only once, whereas males are polygynous, and, although it has a high reproductive potential, field population growth rates are low in tropical areas. In northern areas, eradication was enhanced by cool‐cold weather, whereas eradication in tropical Mexico and Central America is explained by the SIT. Despite low average efficacy of SIT releases (approximately 1.7%), the added pressure of massive SIT releases reduced intrinsically low fly populations, leading to mate‐limited extinction. Non‐autochthonous cases of myiasis occur in North America and, if the fly reestablishes, climate warming by 2045–2055 will expand the area of favourability and increase the frequency and severity of outbreaks.

## Graphical abstract

Deconstruction of the invasion biology of new world screwworm explains the role of North American monsoons with respect to its dynamics and eradication using the sterile insect technique (SIT).
SIT programme myiasis data explained the time lagged effects of weather on observed and potential myiasis outbreaks in Texas. A demographic model explained the fly dynamics in tropical regions.
The efficacy of sterile fly releases was assessed and the effects of climate change on the potential expansion of the fly's geographical range in North America were estimated.

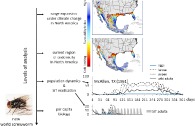

Prelude to the 1972 outbreak

‘*Three tropical storms moved inland along the Texas and northeastern Mexico Gulf Coast in the late summer and autumn of 1971. There was an abundance of rain. The screwworm population began to build up despite releases of 1,140 to 1,520 sterile flies per square kilometer, averaging 135.3 million weekly*’ (Novy, 1991)

## Introduction

The new world screwworm fly *Cochliomyia hominivorax* (Coquerel) is a subtropical‐tropical species of the Americas (Baumhover, 2002; Comis *et al*., 2012; OIE, World Organisation for Animal Health, 2013). Females oviposit in wounds of wildlife, livestock and pets, as well as occasionally humans, and, if untreated, the hatching larvae may cause severe myiasis and death. The fly was eradicated from North America to the Darien Gap in Panama (Wyss, 2000) using releases of large numbers of irradiated sterile flies (i.e. the sterile insect technique, SIT) targeting unmated adult females (Knipling, 1955), establishment of quarantine areas to prevent the introduction of infested animals, and chemical treatment of infested livestock that killed larval stages and possibly adults feeding on serous fluid in wounds (Laake, 1950; Klassen & Curtis, 2005). Eradication efforts began in Florida in 1957 and in Texas in 1962, and subsequently progressed through Mexico during the 1980s to south Panama in the late 1990s (Wyss, 2000), where ongoing containment efforts continue to keep the fly from reinvading northward (Maxwell *et al*., 2017). The last reported autochthonous case of screwworm myiasis in Southwest (SW) U.S.A. occurred in 1982 (Novy, 1991). However, the fly is endemic to the Caribbean and South America (Laake, 1950; Baumhover, 2002; Comis *et al*., 2012) and non‐autochthonous cases of myiasis are occasionally reported in the U.S.A. (USDA‐APHIS, United States Department of Agriculture – Animal and Plant Health Inspection Service, 2017a). Eradication of the fly cost more than 750 million US dollars (Gutierrez & Ponti, 2014), removing an important economic constraint on the livestock sector (Vargas‐Terán *et al*., 2005) and infestations in wildlife. Although not completely attributable to eradication, cattle production in Texas increased from 6.53 million head in 1974 (E. S. Krafsur, personal communication) to 12.32 million in 2016 (USDA, United States Department of Agriculture, 2016).

The present study builds upon the analysis reported by Gutierrez & Ponti (2014) concerning the role of SIT and weather on the eradication of the fly in North America. Specifically, we link the effects of the North American Monsoons (NAMS) to screwworm myiasis outbreaks, and explain why, despite massive releases of sterile adults, outbreaks of myiasis occurred during the 1962–1982 eradication period in the SW U.S.A. (Novy, 1991). We estimate (albeit roughly) the efficacy of SIT releases and map the potential expansion of the fly's geographical range in the U.S.A. under projected climate warming should reinvasion occur.

The biology of screwworm was reviewed and a model of its population dynamics based on available data in the literature was developed by Gutierrez & Ponti (2014); both are only briefly reported here.

### *Biology and migration of screwworm*

Males have a promiscuous mating behaviour (polygyny), whereas females mate only once. This mating biology was a key factor in the eradication success because matings between sterile males and wild females yield non‐viable eggs (Knipling, 1955). Adult male flies feed at flowers and live for 2–3 weeks, whereas adult females live approximately 10 days on average, feeding on serous fluids at animal wounds and decomposing animals (Thomas & Mangan, 1989; OIE, World Organisation for Animal Health, 2013). Approximately 3–4 days after mating, female flies begin to seek wounds on vertebrates to lay large batches of eggs but, because the species is autogenous, females can complete two or three vitellogenic cycles without a protein meal (Crystal, 1966). Under field conditions in Central America, the half‐life of mated wild female is 3.7 days, with a mean age at wounds of 7.5 days and a maximum age of 21 days (Thomas & Chen, 1990).

Screwworm females are attracted to wounds that may be as small as those caused by the feeding of the invasive cattle tick *Rhipicephalus* (*Boophilus*) *microplus* (OIE, World Organisation for Animal Health, 2013), which has periodic outbreaks in Mexico and south Texas (Pérez de León *et al*., 2012). Feeding by screwworm larvae expands the wound (myiasis) attracting further oviposition and, if not treated, this may cause the death of the animal.

### *The effects of temperature*

Screwworm is a cold intolerant species that has high lower and upper developmental thermal thresholds (14.5 and 43.5 °C, respectively) with the optimal temperature for survival and adult reproduction being approximately 27.5 °C (data from Adams, 1979; Berkebile *et al*., 2006). The egg and larval stages develop on/in the host at host body temperature and, at maturity, the larvae drop to the ground to pupate. Pupae and free‐living adults experience near ambient temperatures.

The daily mortality rate of pupae and adults [µ_adult_(*T*)] per day at different temperatures (*T*) was captured by a symmetrical convex function fitted to laboratory data (Adams, 1979; Berkebile *et al*., 2006) (Eqn (1), see Gutierrez & Ponti, 2014).

(1) $$\begin{array}{cc} & 0\leq {µ}_{\text{adult}}\left(T\right)=0.00036\times {\left(T-27.2^{{}^{\circ}}C\right)}^{2}+0.0035\leq 1\\ & \;d.f.=16,r^{2}=0.74 \end{array}$$

In the model, we focus on the limiting effects of average ambient daily temperatures *T* < 27.2 °C that reduce reproduction and survival, especially during the critical autumn–winter period. Temperatures of *T* ≥ 27.2 °C in summer also affect the fly demographic rates, although such temperatures are generally not limiting (Gutierrez & Ponti, 2014). To capture the limiting effects of colder temperatures, the daily values of µ_adult_(*T* < 27.2^°^*C*) were summed over the year [i.e. µ_cold_(y)] (Eqn (2) for each of the 20 335 lattice cells (25 km^2^) across the continental U.S.A. and Mexico during the period 1975–2005.

(2) $${µ}_{\text{cold}}\left(y\right)=\sum_{i=1}^{365\;\text{or}\;366}{µ}_{\text{adult},i}\left(y,T\left(t\right)\leq 27.2^{{}^{\circ}}C\right)$$

**Figure 1:**
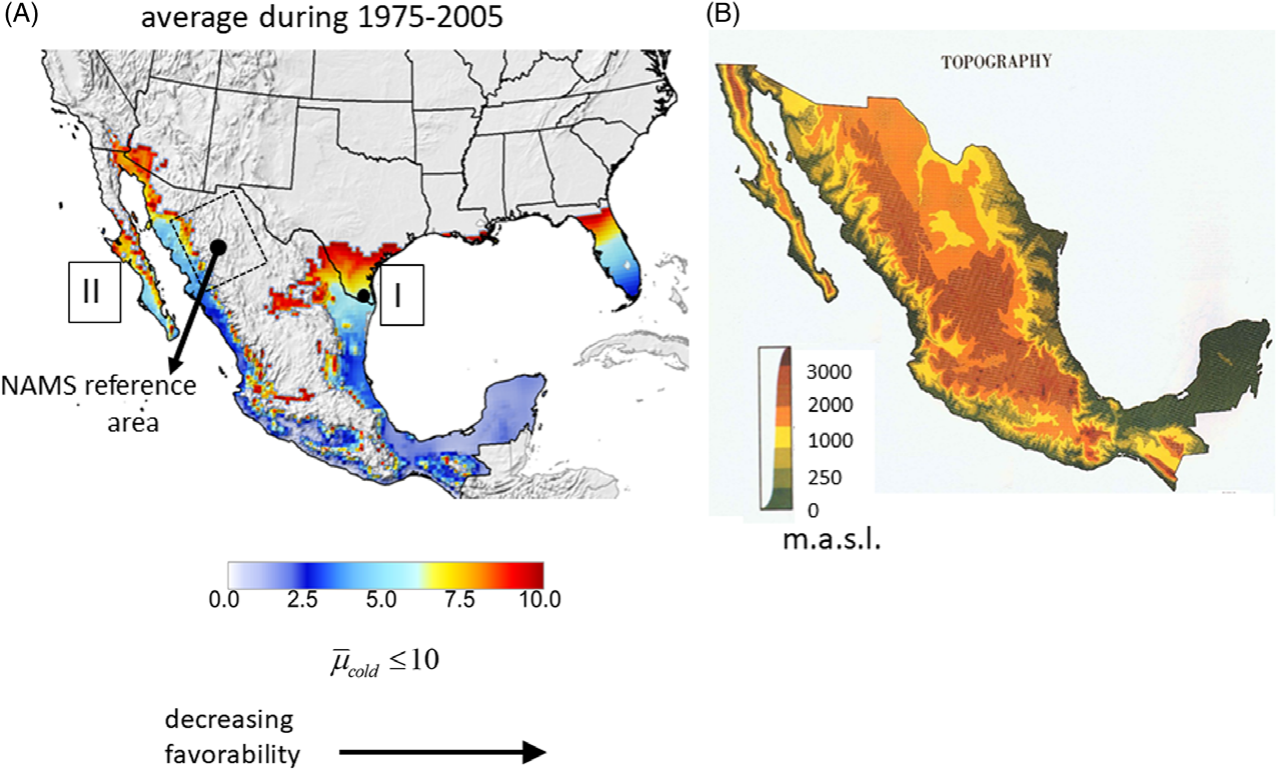
(A) Areas I and II of screwworm endemicity in the U.S.A. and Mexico based on average. ${\bar{µ}}_{\text{cold}}\leq 10$ for the period 1975–2005 [Eqn (2) (Gutierrez & Ponti, 2014)]. Unshaded grey areas have ${\bar{µ}}_{\text{cold}}>$ 10 and are unfavourable. Also shown is the reference area in Northwest (NW) Mexico (dashed rectangle) used to categorize North American Monsoons (NAMS) (Arias *et al*., 2012) and the location of McAllen, Texas (•) in the transition zone of Area I. The topography of Mexico is shown in (B) (https://www.lib.utexas.edu/maps/atlas_mexico/topography.jpg) (m.a.s.l.). [Colour figure can be viewed at http://wileyonlinelibrary.com].

Gutierrez & Ponti (2014) estimated that an average value of ${\bar{µ}}_{\text{cold}}\approx 10$ was a good metric defining the geographical limits for screwworm year round survival (endemicity) that accorded well with field observations. Using this metric, cold weather on average restricts the area of fly endemicity in North America to south Texas and much of Florida, a broad north‐south band of eastern Mexico (the states of Nuevo Leon, Tamaulipas and Veracruz), the Yucatan Peninsula, and tropical south Mexico (Area I), as well as to Baja California and western coastal Mexico (Area II) (Fig. 1A). Higher elevations of Mexico may also be outside of the favourable zone of endemicity (Fig. 1B). We designate the northern reaches of Areas I and II as transition zones of fly persistence.

### *Adult dispersal and migration*

Mated adult females have high disperal capacity, whereas males exhibit an aggregation–wait station behaviour that greatly limits their dispersal (Krafsur, 1978). In the tropics, fly numbers are consistently higher in forest habitats (Phillips *et al*., 2004). In warm humid areas with a high density of animals, dispersal of females is approximately 3 km, although a dispersal of 10–20 km has been reported, with distances of 300 km being documented in arid environments with dispersal aided by prevailing winds that may enable flights across open water (Barrett, 1937; Deonier, 1942; Hightower *et al*., 1965; Mayer & Atzeni, 1993; Skoda *et al*., 2017).

**Figure 2:**
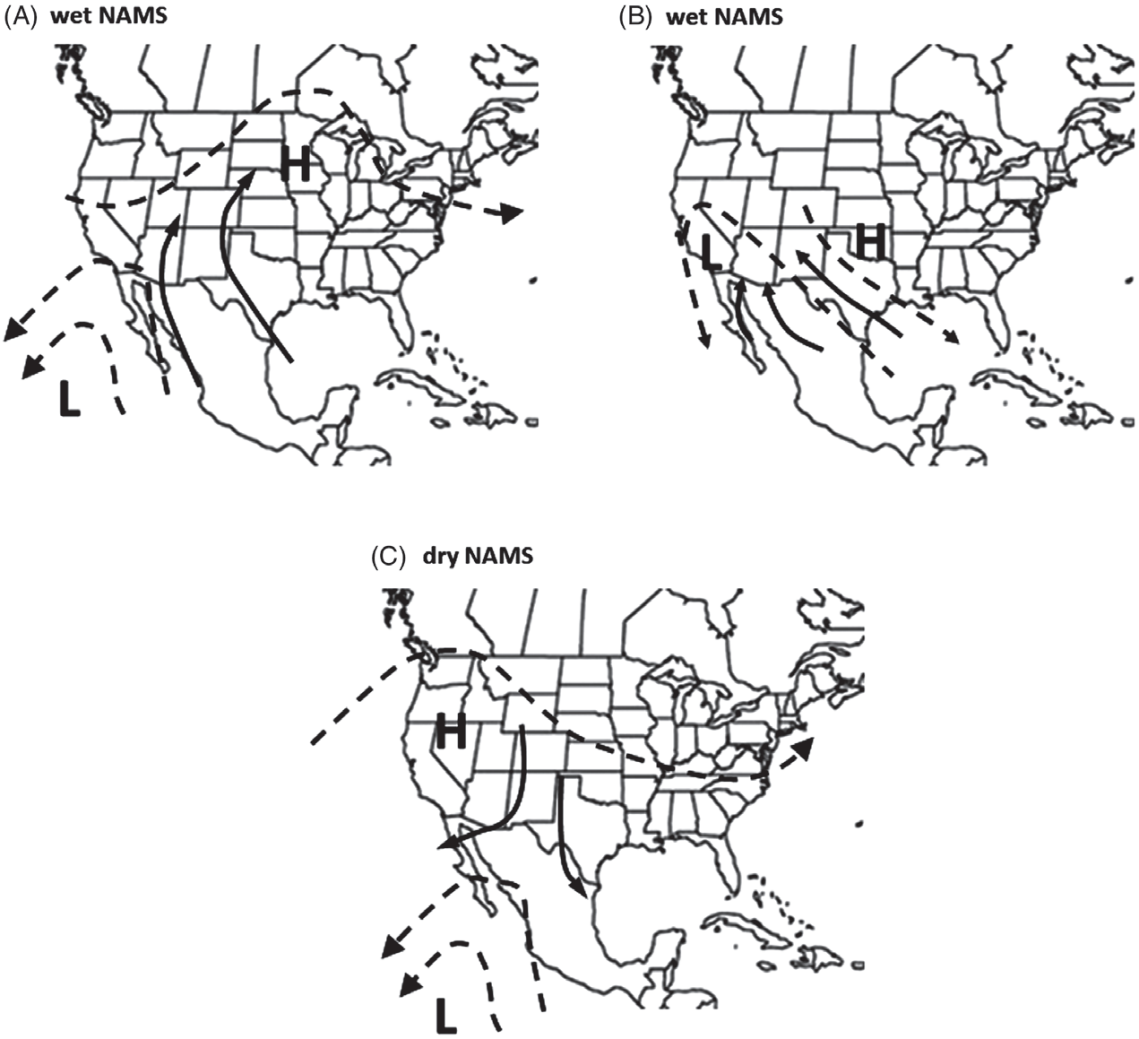
Schematics of typical lower‐troposphere (700 hPa) circulation of air flow (heavy solid arrows line) during: (A, B) wet and (C) dry North American Monsoons (NAMS) events relative to the position of areas of summer high (H) and low (L) surface pressure (maps modified from http://www.cpc.ncep. http://noaa.gov/products/outreach/Report-to-the-Nation-Monsoon_aug04.pdf; http://www.wrh.noaa.gov/twc/monsoon/monsoon_NA.php).

**Figure 3:**
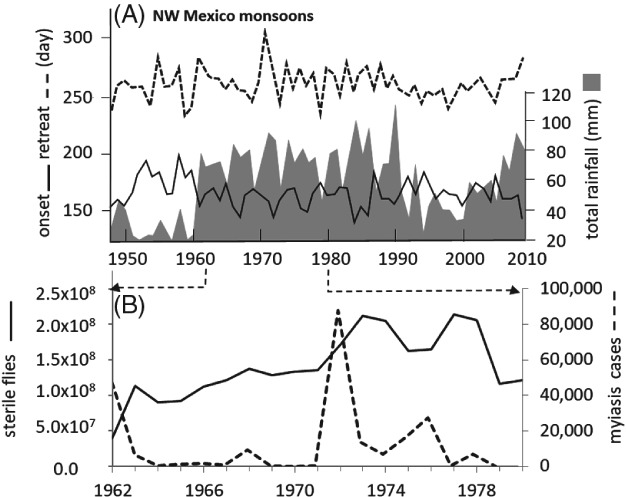
Data on North American Monsoons (NAMS) weather, cases of screwworm myiasis and releases of sterile flies used in the analysis. (A) Julian dates of onset (solid) and retreat (dashed) of annual NAMS and associated rainfall totals in Northwest (NW) Mexico (shaded) during 1948–2010 (Arias *et al*., 2012). (B) Total number of annual cases of screwworm myiasis in the U.S.A. (dashed) and total number of sterile flies released by the sterile insect technique (SIT) eradication programme (solid) (Novy, 1991).

Historically, the first cases of myiasis in the U.S.A. were reported in south Texas (and south Florida) during mid‐winter and, during some years, the infestations spread northward in Texas and neighbouring states but subsided in the autumn as temperatures cooled [Eqn (2) (Baumhover, 2002; Comis *et al*., 2012)]. Reinvasion of non‐endemic areas of the SW U.S.A. during the late spring to autumn period is assumed to be enhanced by NAMS winds (Fig. 2) (Gutierrez & Ponti, 2014). Arias *et al*. (2012) classified NAMS as being wet or dry based on weather in a rectangular area of Northwest (NW) Mexico (20°N 107°W, 22°N 101°W, 32°N 106°W, 30°N 112°W) (Fig. 1A). Although less impressive than the Asian monsoons, wet NAMS regimes (storms) produce northward surges of relatively cool, moist maritime air from the eastern tropical Pacific into the SW U.S.A. via the Gulf of California (Fig. 2A), although some wet NAMS may also draw atmospheric moisture from the Gulf of Mexico (Fig. 2B) (Carleton *et al*., 1990; Douglas *et al*., 1993; Stensrud *et al*., 1995; Adams & Comrie, 1997; Wright *et al*., 2001; Higgins *et al*., 2004; Vera *et al*., 2006). Wide variations of NAMS occur on intra‐seasonal and decadal time‐scales and are related to the frequency of gulf surges and the latitudinal position of the subtropical ridge during the late June to September period. The Julian dates (from 1 January) for the onsets and retreats of NAMS and the associated total amount of monsoon rainfall (mm) during 1948–2010 are shown in Fig. 3A (data from Arias *et al*., 2012). Wet NAMS have early seasonal onset and late retreat, and the prevailing winds are anticyclonic and generally flow in a northward direction. By contrast, dry NAMS have late seasonal onset and early retreats, low rainfall, and prevailing winds are often in a southward direction (Fig. 2C). We use total rainfall in the reference area as a surrogate metric for the strength of annual NAMS, as well as for the dominant direction of storm wind flow favourable for fly movement.

## Methods

### *Myiasis data*

During the SIT programme, larval samples (cases of myiasis) submitted by stockmen during the eradication period (1962–1983) were used to document the geographical distribution and relative levels of myiasis, as well as to target sterile fly releases (E. S. Krafsur, personal communication). Totals of myiasis cases reported in the SW U.S.A. during the 1962–1980 eradication period and the total annual number of sterile insects of both sexes released are shown in Fig. 3B (Novy, 1991; Gutierrez & Ponti, 2014) (see also the ratio data in Fig. SM1 in the Supporting information, File S1). Approximately 96% of the cases were reported from Texas, and hence this area is a major focus of the present study. The myiasis data implicitly include weather and migration effects, stockmen reporting bias, and the effects of SIT releases of hundreds of millions of adult sterile males and females. The myiasis data are monthly county level totals, georeferenced to the county seat, and are mapped as yearly log_10_(*total cases of myiasis* + 1). Data for the 1962 and 1972 outbreaks were further summarized on a weekly and monthly basis respectively, and were mapped to illustrate the time development of the infestations across Texas. The myiasis data are available on request in excel format (Microsoft Corp, Redmond, WA, USA).

### *Weather data*

Observed daily weather data (maximum–minimum temperature and rainfall) for McAllen, Texas for the 1 January 1942 to 31 June 2017 were obtained from the Global Historical Climatology Network – Daily (GHCN‐Daily, Version 3) (Gutzler *et al*., 2005; Menne *et al*., 2012a, 2012b; Arias *et al*., 2012; see supplemental materials, File S1). Daily maximum–minimum temperature and rainfall data for the historical period 1975–2005 and for the future period 2045–2075 at each of 20 355 lattice cells of approximately 25 km^2^ (spatial resolution 0.25°) across the U.S.A. and Mexico were obtained from the National Aeronautics and Space Administration (NASA) Earth Exchange Global Daily Downscaled Projections (NEX‐GDDP) dataset (Thrasher *et al*., 2012; https://nex.nasa.gov/nex/projects/1356/). The NEX‐GDDP dataset includes global downscaled climate scenarios that are derived from the General Circulation Model (GCM) runs conducted under the Coupled Model Intercomparison Project Phase 5 (CMIP5) (Taylor *et al*., 2012). Specifically, we use climate model data from the Max Planck Institute Earth System Model low resolution (MPI‐ESM‐LR) GCM, forced by the Representative Concentration Pathway 8.5 (RCP 8.5), a scenario of high greenhouse gas emissions relative to other RCPs (Riahi *et al*., 2011), as downscaled in the NEX‐GDDP dataset. Sheffield *et al*. (2013) evaluated historical simulations of North American climate in CMIP5 experiments using continental metrics of bias relative to weather observations and showed that MPI‐ESM‐LR is the top ranked among the core set of 17 GCMs considered, with a particularly good performance in terms of temperature. The NEX‐GDDP dataset also provides a set of global, high resolution, bias‐corrected climate change projections for use when assessing climate change impacts on processes that are sensitive to finer‐scale climate and local topography, including biological processes of poikilotherm organisms such as screwworm. Downscaling (i.e. increasing the spatial resolution) of GCM output addresses two primary limitations: the relatively coarse spatial resolution of most GCMs (e.g. hundreds of km) and their statistical bias compared with observations (Thrasher *et al*., 2012).

## Results

Our analysis initially focuses on the dynamics of myiasis outbreaks in Texas, and subsequently explores the dynamics of SIT eradication in tropical Mexico.

### *Analysis of myiasis outbreaks in Texas*

Despite massive releases of sterile flies, five peaks of myiasis of varying intensity occurred during the 1962–1982 eradication period: 1962, 1968, 1972, 1975–1976 and 1978 (Fig. 3B). A small outbreak in 1978 was mostly a result of cases from Arizona and New Mexico. Prior analysis of the same myiasis data (Gutierrez & Ponti, 2014) suggested that cold weather and total rainfall at McAllen, Texas during the previous year (*y −* 1) could be used as predictors of screwworm myiasis outbreaks in Texas during the current year (*y*).

**Figure 4:**
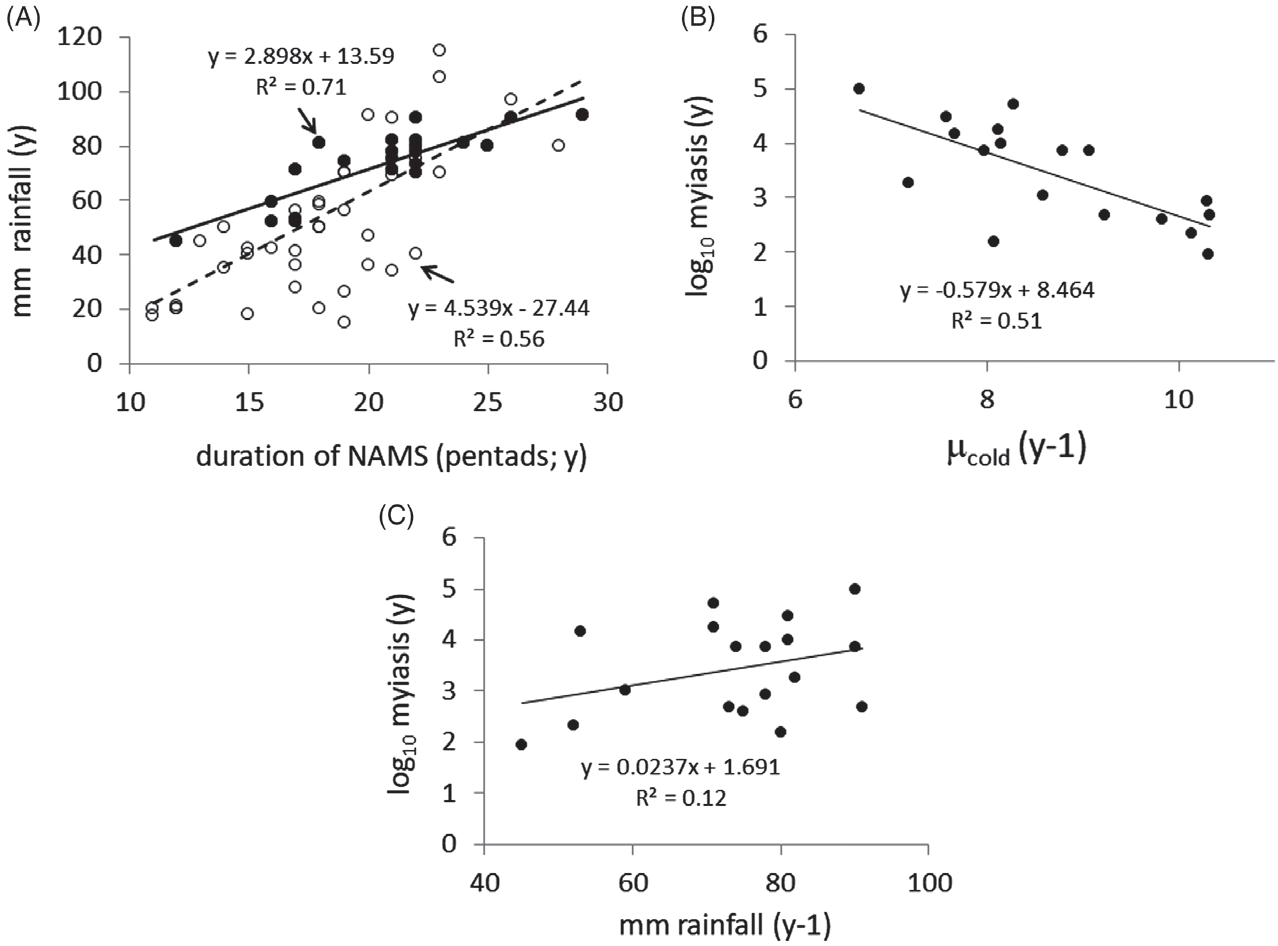
Linear regressions of factors affecting screwworm myiasis. (A) Total monsoon rainfall on the duration of North American Monsoons (NAMS) in pentads (5‐days increments) in Northwest (NW) Mexico (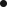, 1961–1983; 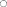 and 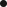, 1948–2010; data from Arias *et al*., 2012). (B) Regression of log_10_ cases of myiasis in year *y* in the Southwest (SW) U.S.A. on cumulative daily mortality rates (µ_cold_(*y* – 1)) and (C) log_10_ cases of myiasis in year *y* on total rainfall in NW Mexico in year *y* – 1. The screwworm data are from Novy (1991) and Gutierrez & Ponti (2014).

In the present study, log_10_ cases of myiasis(*y*) in year (*y*) were regressed on (a) the dates in five‐day intervals (pentads) of the onset and retreat of NAMS; (b) their duration; (c) total rainfall (mm) in the NW Mexico reference area (data from Arias *et al*., 2012) (Fig. 2A); (d) yearly estimates of µ_cold_ at McAllen, Texas, as a measure of temperature conditions in the north transition zone (Area I); and (e) yearly log_10_ sterile insects released. We note that total rainfall in NW Mexico during the eradication period is correlated to the duration of NAMS in pentads (*r* ^*2*^ = 0.71) (Fig. 4A), although the relationship for the 1948–2010 period is weaker (*r* ^*2*^ = 0.56).

Multiple linear regression analysis (Eqn 3) found that only lagged values of µ_cold_ (*y* − 1) at McAllen in the transition zone, and total rainfall (*y* − 1) in the NW Mexico reference area were significant predictors of log_10_ cases of myiasis(*y*). None of the interaction terms were significant, and the correlation between µ_cold_ (*y*) and total rain (*y*) was weak (*r* ^*2*^ = 0.053). This regression model has the added advantage (Gutierrez & Ponti, 2014) in that rainfall values suggest the strength of the NAMS and the direction of the prevailing monsoon winds (Arias *et al*., 2012).

(3) $$\begin{array}{cc} & {\text{log}}_{10}\text{myiasis}\left(y\right)=6.568+0.028\;\text{rain}\left(y-1\right)\\ & -0.602{µ}_{\text{cold}}\left(y-1\right)\;\;r^{2}=0.63,F=12.63,d.f.=15 \end{array}$$

$$\begin{array}{cc} & \text{mean values}\;\left(\text{coefficient}\;t‐\text{values}\right)\\ & =\left\{\begin{array}{l} \log_{10}\text{myiasis}\left(y\right)=3.433\\ {µ}_{\text{cold}}\left(y-1\right)=8.69\;\left(-4.71^{**}\right)\\ \text{rain}\left(y-1\right)=75.50\;\left(2.16^{*}\right)\\ {}^{**}\;P<0.01,{}^{*}\;P<0.05 \end{array}\right. \end{array}$$

log_10_ *myiasis*(*y*) declines with increasing µ_cold_(*y* − 1) (*P* < 0.01) and increases with total rainfall (*y* − 1) (*P* ≈ 0.05) (Fig. 4B,C).

Substituting values of cumulative µ_cold_(*autumn–winter*) computed for the period 1 September to 31 May that bridges 2 years (i.e. *y −* 1 and *y*) for µ_cold_(*y* − 1) in the multiple regression yielded a low predictive value (*r* ^*2*^ = 0.287), with only µ_cold_(*autumn–winter*) being significant (*P* < 0.05). This suggests that screwworm buildup occurs in Area I during the prior year (*y − 1*) and that conditions during the preceding winter period simply set the stage for outbreaks during summer in Texas in year *y*.

### *Favourability of weather for myiasis outbreaks during 1948–2010*

**Figure 5:**
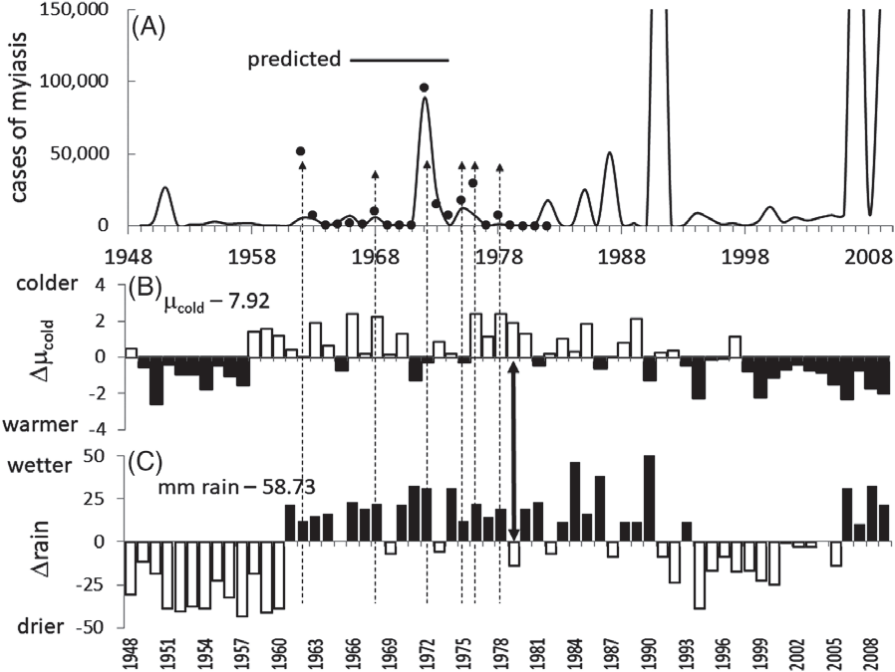
Screwworm dynamics and North American Monsoons (NAMS) characteristics. (A) observed cases of screwworm myiasis (data symbol 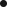) during 1962–1983 (Novy, 1991; Gutierrez & Ponti, 2014) and simulated cases using Eqn 3 during 1948–2010 (solid line). (B) deviations from average cumulative daily mortality rates (i.e. Δµ_cold_ = µ_cold_(*y*) − 7.92) at McAllen TX during 1948–2010 (Gutierrez & Ponti, 2014). (C) Deviations from average total annual rainfall (i.e. Δ*rain* = mm rainfall(*y*) − 58.73 mm) in the NAMS reference area in Northwest (NW) Mexico during 1948–2009 (data from Arias *et al*., 2012). Deviations from average values for µ_cold_ and rainfall that are favourable for screwworm outbreaks are shaded black. The dashed arrows point to observed outbreaks, and the solid double arrow points to the critical year 1979 (see text).

The data used in estimating Eqn (3) include the effects of weather, the vagaries of stockman bias and the non‐significant effects of log_10_ sterile insect released, and may be viewed as an ecological niche model for screwworm outbreaks in Texas. Using Eqn (3), we examined the favourability of weather for myiasis outbreaks in Texas before (1948–1961), during the SIT eradication period (1962–1982) and after (1983–2010). Observed and predicted total myiasis are shown in Fig. 5(A). During 1948–2010, average ${\bar{µ}}_{\text{cold}}$ at McAllen was 7.92 ± 1.28 per year and average rainfall in the NW Mexico reference area was 58.73 ± 24.82 mm/year. Annual deviations from these averages (i.e. *Δ*µ_cold_ and Δ*rain*) are depicted as histograms in Fig. 5B,C, with deviations favourable for screwworm outbreaks indicated in black.

#### *1948–1961 period*

During the pre‐SIT 1948–1960 period, NAMS were drier than average with prevailing winds in a southerly direction. µ_cold_ values during 1948 to 1957 were relatively low, suggesting the potential for higher‐than‐average winter survival and also that cases of myiasis would have occurred in the south Texas transition area. By contrast, years 1958–1960 were cold with low predicted screwworm survival. An outbreak of myiasis is predicted only in 1951 as a result of a warmer than average 1950 and a NAMS with slightly below‐average rainfall [i.e. the year lag effect in Eqn (3)].

#### *1962–1982 period*

As expected, the observed and predicted values (Eqn 3) of outbreaks during this period are in reasonable agreement (*y* = 0.74*x* − 1049.4; *r*^2^ = 0.75), with only the 1962 value being a 10× outlier (Fig. 5A). The 1962 outbreak of 51.6 thousand cases of myiasis was preceded in 1961 by near average µ_cold_ (= 8.31) and an above average wet NAMS (80 mm) with a late retreat on October 10 and with prevailing winds in a northward direction. Examination of the 1961–1962 weather data revealed no anomalies that could explain the outlier that was posited as a result of delays in sterile insect releases (Krafsur & Garcia, 1978), although over reporting during the initial year of the SIT programme may have been a factor (see Fig. SM2A in the Supporting information, File S1). The small outbreak in 1968 was preceded in 1967 by near‐average µ_cold_ and a modest wet NAMS. By contrast, the large 1972 outbreak of 95.6 thousand cases was preceded in 1971 by lower‐than‐average µ_cold_ = 6.67 (i.e. good survival) and an above‐average wet NAMS (approximately 80 mm) with northward prevailing winds and a very late retreat in late October. The extent of the 1972 outbreak in the U.S.A. and Mexico is summarized in Fig. SM2B in the Supporting information, File S1. The small 1975 and 1976 outbreaks were preceded by years of near‐average µ_cold_ (7.5 and 8.1, respectively) and above‐average wet NAMS with moderately late retreats in 10 and 30 September, respectively.

Years 1976, 1977, 1978 and 1980 had moderately wet NAMS but experienced cold weather (µ_cold_ = 10.3, 9.1, 10.3 and 9.2, respectively), whereas 1979 was very dry (approximately 48 mm) and cold (µ_cold_ = 9.8) and only low scattered infestations occurred in Texas (see maps of all years, Fig. SM3 in the Supporting information, File S1). The cold period 1976–1980 reduced screwworm populations that coupled with massive sterile insect releases enabled eradication of the fly in the transition zone of Area I. This assertion is reinforced by the fact that, despite favourable weather during 1981 (µ_cold_ = 7.49) and a strong NAMS (82 mm rainfall), the predicted outbreaks in 1982 and in subsequent years did not occur (see below). The last autochthonous case of myiasis in the U.S.A. was recorded in 1982, enabling expansion of the SIT programme into Mexico (Novy, 1991; Wyss, 2000).

#### *1983–2010 period*

Had eradication not occurred in the transition zone, weather conditions were highly favourable for outbreaks in Texas during some years of the 1983–2010 period. The 1990 NAMS was wet and µ_cold_ was below‐average and a very large outbreak is predicted for 1991. From 1991 to 2005, the NAMS were dry but µ_cold_ was consistently favourable, and small outbreaks were predicted. Wet NAMS with late retreats and favourable below‐average µ_cold_ occurred during 2006–2009, and large outbreaks were predicted in 2007, 2009 and 2010 but did not occur (Fig. 5A). Absent eradication, these favourable conditions could have enhanced fly outbreaks that would have overwhelmed extant eradication efforts as occurred in 1972.

### *Eradication of screwworm in tropical areas of Mexico*

Myiasis data from Mexico are not available, and Eqn (3) for Texas cannot be used to examine the favourability of weather for myiasis outbreaks in this region. Instead, we used an age‐structured, physiologically‐based demographic model (PBDM) (Gutierrez & Ponti, 2014) to capture the fly's weather driven dynamics and to explore SIT eradication of the fly in Mexico. The bio‐demographic functions for development, survival and reproduction in the PBDM depend on temperature, and additionally oviposition site density for reproduction (see below). Although Thomas (1993) asserted that ‘… *evidence is lacking that laboratory‐adapted strains are competitively impaired in the field* …, *laboratory adapted strains clearly deviate from the wildtype*’ questions arose whether reproductively isolated population could impede the progress of the eradication campaign (Richardson *et al*., 1982), whether sterile flies were competitive with wild ecotypes (Bush *et al*., 1976) and whether aerial fly release procedures were efficient (Krafsur & Garcia, 1978; Krafsur, 1987). Lachance *et al*. (1982) found that allelic frequencies were similar in wide geographically separated populations and no genetic mating incompatibilities were found. Data to estimate the effects of competitiveness and release procedure are not available, although a very rough estimate of their combined effect can be made heuristically by comparing observed field release rates with base PBDM predictions of sterile fly release rates required for eradication, assuming sterile flies have the same mating competitiveness, and the release procedures are non‐limiting.

Absent SIT effects, the number of fertile eggs (*ΔE*(*t*,*T*)) deposited by all fertile mated females (*W*_*m*_) and newly mated females (0.5*W*_*u*_) at time *t* (i.e. day) is computed using Eqn (4) (Gutierrez *et al*., 2014).

(4) $$\begin{array}{cc} & \Delta E\left(t,T\right)=\phi_{T}\left(T\left(t\right)\right)\phi_{\mathrm{lx}}\left(T\left(t\right)\right)\phi_{\text{search}}\left(H,T\left(t\right)\right)\\ & \cdot \mathrm{sr}\cdot R\cdot \left(W_{m}\left(t\right)+0.5W_{u}\left(t\right)\right) \end{array}$$

Simplifying the notation:

(4i) $$\Delta E=\phi_{T}\phi_{\mathrm{lx}}\phi_{\text{search}}\cdot \mathrm{sr}\cdot R\cdot \left(W_{m}+0.5W_{u}\right)$$

Only half of virgin females are mated per day (Krafsur, 1985), and the average maximum per capita reproductive rate per female per day *R* is approximately 67 eggs/days. This potential reproduction is corrected for sex ratio (*sr* = 0.5) and scaled by concave symmetrical functions for the effects of temperature on reproduction (0 ≤ ϕ_T_(*T*(*t*)) ≤ 1, 14.5 °C *≤ T ≤* 43.5 °C; estimated from Thomas & Mangan, 1992) and for adult survival (0 ≤ ϕ_lx_(*T*) < 1) (Gutierrez *et al*., 2006). A ratio‐dependent functional response model ($0\leq \phi_{\text{search}}\left(t,T\right)=\left(1-e^{-0.0001\mathrm{\Delta t}\left(T\left(t\right)\right)\cdot H/W_{m}\left(t\right)}\right)<1$) (Gutierrez & Baumgärtner, 1984) was used to estimate the success of fertile females in finding wounds (*H* km^−2^) given a low search rate (0.0001 per dd) where Δ*t*(*T*(*t*)) is physiological time in degree days > 14.5°C (dd) on day *t*. In the field, *H* varies in time and space in unknown ways (Matlock *et al*., 1996; Matlock & Skoda, 2009) and hence a constant (*H* = 100) was used for comparative purposes.

SIT affects only unmated females and the effects enter the model Eqn (4) as scalar functions for mating competitiveness (0 < ϕ_comp_ < 1) and, as a linear scalar (0 < ϕ_release_ < 1) for the relative efficacy of sterile fly releases for mating with virgin females (Eqn 5). The ϕ functions can be viewed as survivorship terms.

(5) $$\;\Delta E=\phi_{T}\phi_{\mathrm{lx}}\phi_{\text{search}}\mathrm{sr}\cdot R\cdot \left(W_{m}+0.5\cdot \phi_{\text{comp}}\phi_{\text{release}}W_{u}\right)$$

Specifically, 0 < ϕ_comp_ = *W*_♂_/(*cS*_*♂*_ + *W*_*♂*_) ≤ 1 is the proportion of *W*_u_ mated by wild type males (*W*_*♂*_) competing with sterile males (*S*_*♂*_) with coefficient 0 < *c* ≤ 1 being the competitiveness of *S*_*♂*_ relative to *W*_*♂*_ (Krafsur, 1994). Hence, 0.5ϕ_comp_*W*_u_(*t*) is the number of *W*_u_ × *W*_*♂*_ matings, and 0.5(1 − ϕ_comp_)ϕ_release_*W*_u_(*t*) is the number of *W*_u_ × *S*_*♂*_ matings removing females from the reproductive pool. The remaining unmated females is *W*_u_(*t* + 1) = 0.5*W*_u_(*t*) + *ΔW*_u_(*t*), where Δ*W*_*u*_(*t*) is the new females emerging from pupae during *t*.

### *Estimating the efficacy of SIT*

**Figure 6:**
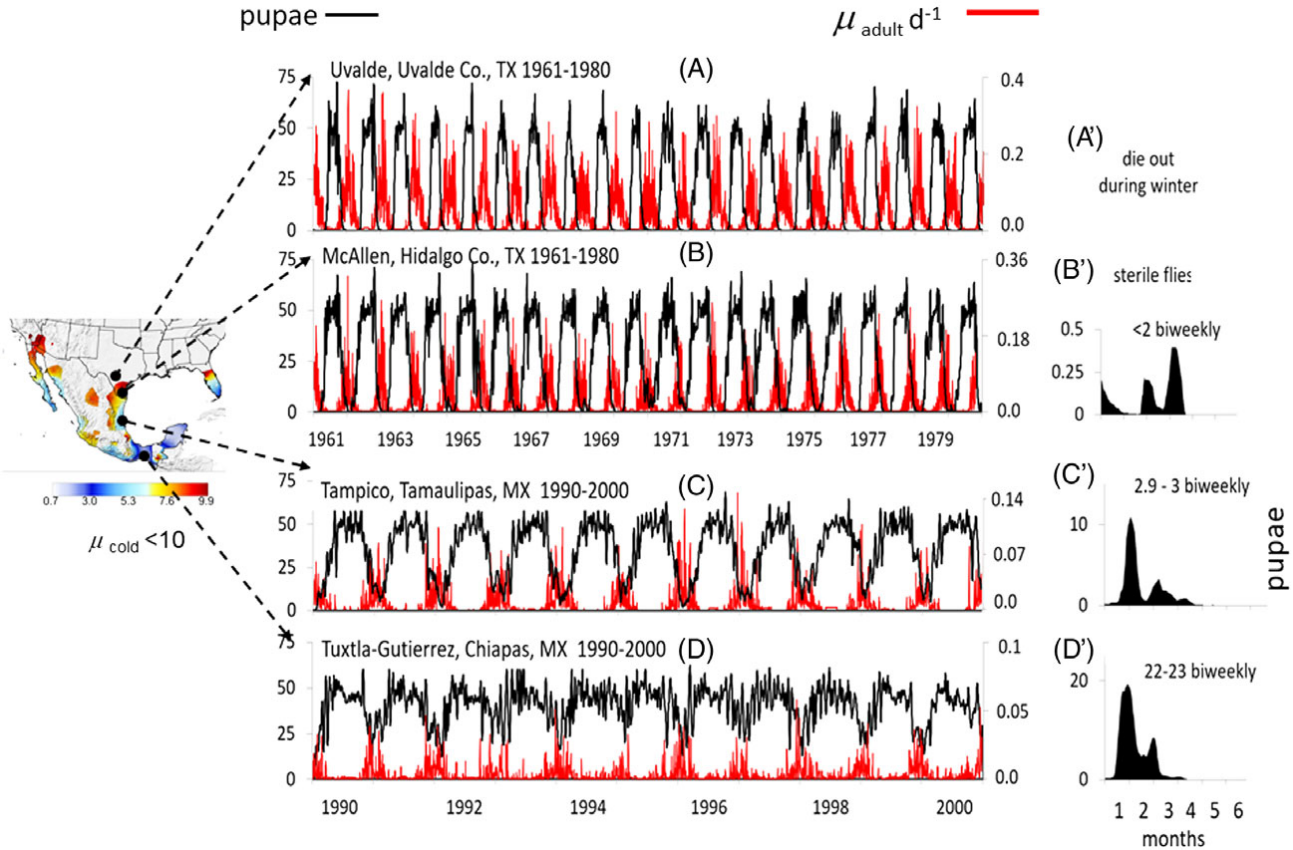
Simulated screwworm pupal numbers in four locations using 1961–2000 weather. (A–D) Simulation of screwworm pupal dynamics (black line, left scale) in the absence of eradication at the four locations using initial densities of 0.25 km^−2^ for all life stages, whereas the solid red line is the daily pattern of µ_adult_ (right scale). Note the large difference in the scale for µ_adult_ across locations. The right most solid black graphs (B′–D′) are the eradication dynamics based on the indicated number of bi‐weekly releases of sterile flies using 1961 weather data for Uvalde and McAllen, Texas, and 1990 data for Tampico and Tuxtla‐Gutierrez, Mexico. [Colour figure can be viewed at http://wileyonlinelibrary.com].

The daily population dynamics of screwworm without and with SIT were simulated at four distinct ecological locations on a north–south transect: Uvalde, Uvalde County and McAllen, Hidalgo County, Texas, and at Tampico, Tamaulipas and Tuxtla‐Gutierrez, Chiapas, Mexico (Fig. 6). Observed daily weather at each location was used to drive the model: years 1961–1980 for Uvalde and McAllen and 1990–2000 weather for Tampico and Tuxtla‐Gutierrez. As initial conditions, *H* = 100, and 0.25 individuals of both sexes of each life stage km^−2^ were used, yielding the relative levels of flies observed in the field (Matlock *et al*., 1996). Simulated pupal numbers was used as the metric of fly density.

The efficacy of SIT was estimated heuristically at the four locations by comparing observed field release rates to PBDM predictions of release rates required for eradication assuming no detrimental effects on sterile flies. Specifically, *W*_u_ × *S*_*♂*_ mating is assumed to be frequency dependent (i.e. 0 < ϕ_comp_ *= cS*_*♂*_/(*cS*_*♂*_ + *W*_*♂*_) ≤ 1, given *c* = 1) and the released sterile flies are assumed to be optimally placed (ϕ_release_ = 1) in proximity to wild virgin females. The bi‐weekly release rate of sterile flies was varied in the different simulations until all screwworm life stages decline to zero.

Uvalde, Texas is located north of the transition zone, and has historically experienced high incidence of myiasis (e.g. Figs SM2 and SM3 in the Supporting information, File S1). During late spring and summer, simulated populations grow rapidly (Fig. 6A) but, as the autumn temperatures cool, reproduction and survival decline to zero (µ_adult_) (Fig. 6A) requiring annual reinitialization of fly populations in the simulation. Annual µ_cold_ (i.e. the sum of daily µ_adult_) during 1961–1983 ranged from 13–18, leading to local extinction of the fly, thus obviating the need for local eradication efforts.

**Figure 7:**
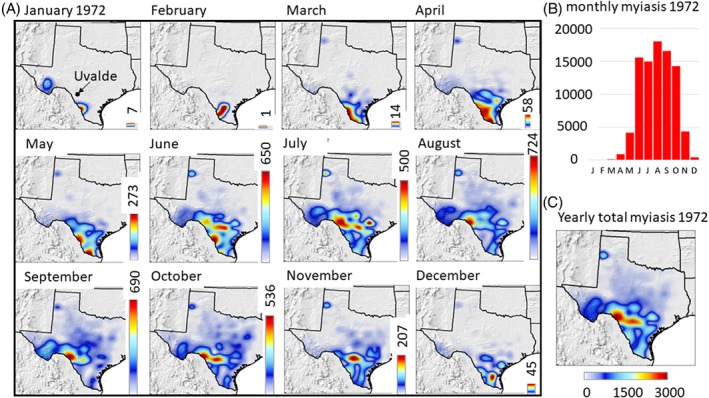
(A) Monthly maps of observed cases of myiasis in Texas during the 1972 outbreak. Highest abundance occurred in August, and the length of all other density colour bars were scaled to it (bars in right hand margin of each map). The maximum density is at the top of each colour bar with the highest midseason incidence occurring in south central Texas (e.g. Uvalde County). (B) Histogram summarizing the monthly total statewide data. (C) Map of total cases of myiasis during 1972. [Colour figure can be viewed at http://wileyonlinelibrary.com].

McAllen, Texas in the transition zone had average µ_cold_ of 7.92, allowing low level overwinter survival, although cold temperatures during 1979 caused near local extinction (see above) (Figs 5B and 6B). In the model, bi‐weekly releases of two sterile flies km^−2^ (i.e. both sexes) were sufficient for eradication (Fig. 6B′); a level grossly at odds with the observed weekly release rates of 39–896 sterile flies km^−2^ (Matlock *et al*., 1996). During the 1972 outbreak, 1890 sterile flies were released per case of myiasis (Fig. 3B) (see also Fig. SM1 in the Supporting information, File S1) and yet the number of reported cases in Texas remained at approximately 16 500 per month during summer (Fig. 7B). Using the simulated average daily density across all years (e.g. 12.67 adult flies km^−2^/day) (Fig. 6B) as the initial density, bi‐weekly releases of 15 sterile flies were required for simulated eradication (see Fig. SM5 in the Supporting information, File S1).

In tropical areas such as Tampico and Tuxtla‐Gutierrez, Mexico, fly populations are endemic and, despite periods of cool temperatures, local extinction is unlikely (Fig. 6C,D). Simulated fly populations at Tuxtla‐Gutierrez show less variability than at Tampico where winter temperatures are cooler during winter. The effects of cool temperatures in this region are seen in field data from Pozo‐Rico, Veracruz, Mexico, where oviposition rates fell to zero and adult mortality increased as temperatures declined to near the developmental threshold of 14.5 °C (see Fig. SM6A,C in the Supporting information, File S1) (data from Krafsur *et al*., 1979). Simulated eradication at Tampico required bi‐weekly releases of approximately 3 sterile flies/km^2^, whereas 23 sterile flies were required at Tuxtla‐Gutierrez (Fig. 6C′,D′). The simulated approximately 7.7‐fold difference is a result of cooler weather at Tampico. The low simulated biweekly values contrast sharply to with weekly field releases of 386 sterile flies/week/km^2^ in Mexico (Krafsur, 1985). The observed weekly release‐rate at Tuxtla‐Gutierrez was approximately 33.5‐fold higher than the simulated bi‐weekly values for a comparative efficacy of 3%. The low field efficiency of SIT is further shown by the modest sterile mating rates that ranged from 10% to 31% in Mexico (Krafsur *et al*., 1979, 1980) and from 18.6% to 71.4% in Mexico and Guatemala (Krafsur *et al*., 1987); with this at low native fly densities (e.g. 7.2 to 53.9 females/km^2^ in Mexico). The efficacy of SIT releases corrected for mating success (e.g. 18.6–71.4% above) was computed as the percentage of unmated females × 0.03, yielding an efficacy range of 0.1–2.4% with an average of approximately 1.7%. These values serve to inform qualitatively why massive numbers of sterile males were required to eradicate relatively low field populations. Furthermore, field evidence suggests that the effect of ϕ_release_ ≫ ϕ_comp_ (Krafsur & Garcia, 1978; Krafsur,1987).

## Discussion

Before eradication, the invasion of screwworm into non‐endemic temperate areas of Texas and the SW U.S.A. during some summers was similar to that of bushfly *Musca vetustissima* Walker, which annually reinvades colder areas of Southeast Australia from warmer inland areas on prevailing winds during spring, only to die out during winter (Hughes & Nicholas, 1974). The invasion biology of screwworm differs in that there is a year lag for outbreaks to develop in Texas. Specifically, screwworm adults must migrate northward into the transition area of NE Mexico and south Texas during summer–early autumn the previous year (*y* − 1) on wet NAMS winds from warmer more southern endemic areas of Mexico. If temperatures during autumn–winter (*y* − 1) are favourable for adult survival and reproduction, the populations may grow and, aided by monsoon winds, spread northward during summer of year *y*. Despite massive releases of sterile flies, favourable weather conditions enabled myiasis outbreaks to develop in 1962, 1972, 1975–1976 and 1978 (Fig. 5; see also Fig. SM1 in the Supporting information, File S1). An additional undocumented risk factor enhancing the outbreaks may have been the increase in cattle tick populations (and associated wounds) in the Rio Grande River area in the south Texas transition zone beginning in 1960 (Pérez de León *et al*., 2012), with outbreaks that appear to mirror those of screwworm during the period (see Fig. SM4 in the Supporting information, File S1).

The outbreaks in 1962 (see Fig. SM2 in the Supporting information, File S1) and 1972 (Fig. 7A–C) were especially large (Fig. 5), with initial infestations first being detected in south Texas during January, increasing in the spring and summer, and spreading northward into Texas during the summer but then retreating to low levels in south Texas during autumn and winter (Fig. 7B). The massive 1972 outbreak also engulfed much of Northern Mexico (see Fig. SM2B in the Supporting information, File S1). The highest levels of myiasis occurred in central and south Texas (e.g. Uvalde), with few cases occurring in the eastern and northern parts of the state (Fig. 7C; see also Fig. SM3 in the Supporting information, File S1). However, with an absence of favourable NAMS in year *y* − 1, warm autumn–winter weather (low µ_cold_) during year *y* − 1 could still enable localized infestations to develop in south Texas (Fig. 5A–C; see also Fig. SM3 in the Supporting information, File S1).

Readshaw (1986, 1989) focused on the role of cold weather during 1976–1980 with respect to suppressing screwworm and concluded that the eradication of the fly was a ‘grand delusion’ and that outbreaks would reoccur once favourable conditions returned. This idea was obviously mistaken because no autochthonous cases of myiasis were recorded in the U.S.A. after 1982 (Krafsur *et al*., 1986; Krafsur, 1998), despite years with highly favourable conditions for outbreaks (e.g. 1992, 2007, 2009 and 2010) (Fig. 5). However, the period 1976–1979 appears to have been critical for eradication because cold weather and weak NAMS (Fig. 5) suppressed screwworm populations and greatly increased the ratios of sterile flies to wild flies (i.e. 10^5^–10^6^ sterile flies released annually per case of myiasis) (see Fig. SM1 in the Supporting information, File S1).

Because records of myiasis and sterile insect releases are not available from Mexico and Central America, we used a physiologically based demographic model to explore heuristically the role of weather and screwworm life history on eradications in the tropics.

### *Role of screwworm life history and weather in fly eradication in Mexico‐Central America*

Ecologists venture that adaptive life histories strategies evolve as a consequence of density and environmental factors (Oizumi *et al*., 2016). Indicative of an r‐strategy (Pianka, 1970) in screwworm are its very high reproductive potential, allowing rapid opportunistic exploitation of oviposition sites (Thomas & Mangan, 1989), a potential population doubling times of 14 days (Matlock & Skoda, 2009), and an aggregating behaviour of screwworm males and low dispersal of unmated females that enhance mating before dispersal (Krafsur, 1978; Krafsur & Garcia, 1978), as well as promiscuous mating in males (polygyny) and single mating in females. The r‐strategy potential of screwworm is demonstrated by outbreaks during some years induced by low densities of mated female migrants invading non‐endemic temperate areas of Texas.

However, in the tropics, growth rates of endemic field population are low (Thomas & Mangan, 1992) with field doubling times ranging from 54 to 139 days (Matlock & Skoda, 2009), oviposition site densities (wounds) are low and can be viewed as an environmental carrying capacity (Krafsur *et al*., 1979), and there appears to be a low innate success rate in finding oviposition sites (Krafsur *et al*., 1979, Krafsur *et al*., 1980). These attributes in the tropics in addition to promiscuous mating in males (polygyny) and single mating in females makes screwworm highly susceptible to massive SIT releases, this despite an estimated low average efficacy of approximately 1.7%.

Furthermore, screwworm has boom to bust dynamics (Krafsur, 1998) as the oviposition sites and weather allow (Krafsur *et al*., 1980). Bust phases occur with declining temperatures that decrease fly vital rates and increase mortality rates (e.g. McAllen, Texas, and Tampico, Mexico), suggesting that the added load of massive sterile fly releases during bust periods, even if not all females are sterile mated, could drive intrinsically low screwworm populations to demographic mate limited ‘Allee’ extinction (Courchamp *et al*., 2008). In tropical areas such as Tuxtla‐Gutierrez, cold weather effects are weak (Fig. 6D) and hence higher simulated levels of SIT releases were required compared with Tampico where low non‐freezing temperatures occur (Fig. 6C). The contrasting simulated dynamics of screwworm at McAllen and Tuxtla‐Gutierrez are shown in Fig. SM5 in the Supporting information, File S1.

By contrast with screwworm, a climatically adapted species such as olive fly with high reproductive rates, abundant hosts and other r‐selected attributes has proven difficult to eradicate using SIT methods. This may have been a result of asynchronous mating activity between the wild and released sterile populations, and/or low competitiveness of the radiation‐sterilized mass‐reared flies (Ant *et al*., 2012). SIT eradication efforts of pink bollworm in the SW U.S.A. were coupled with the highly effective GMO Bt cotton that greatly reduces wild populations (http://www.westernfarmpress.com/cotton/pink-bollworm-eradication-cotton-2017). However, claims of SIT eradication of pink bollworm in the San Joaquin Valley of central California are flawed because the moth cannot overwinter there and Bt cotton is not widely grown (Gutierrez *et al*., 2006). In general, claims of establishment of invasive species below detectable levels or of eradication (e.g. fruit flies) (Papadopoulos *et al*., 2013) are suspect if the effects of weather on the dynamics are ignored.

### *Potential reinvasion and prospective effects of climate change*

Screwworm is endemic to the Caribbean and South America, and periodic cases of myiasis occur in North America (Alexander, 2006) as exemplified by the severe outbreak of myiasis in deer (*Odocoileus virginianus clavium*) populations in the Florida Keys in 2016. This infestation was eradicated by releasing 188 million sterile flies (USDA‐APHIS, United States Department of Agriculture – Animal and Plant Health Inspection Service, 2017a; Skoda *et al*., 2018). SIT containment of the fly continues in Panama, and the early detection and control of sporadic infestations in North America remains the mainstay of screwworm management. In 2000, USDA‐APHIS began producing millions of sterile flies annually in its Panama production facility, and releasing them over eastern Panama and areas of Colombia at a cost of $15 million annually (USDA‐APHIS, 2017b). Despite this effort in Panama, ‘*… an increased number of* [myiasis] *cases in … clusters could be due to SIT failure, the regular transport of screwworm‐positive animals …, movement of screwworm‐positive wildlife and a lack of fly control in neighboring Colombia*’ (Maxwell *et al*., 2017). Such problems are potential harbingers of difficulties that may be encountered if attempts are made to extend eradication across the vast tropical and subtropical areas of South America (Gutierrez & Ponti, 2014); a problem that may be exacerbated by climate change.

**Figure 8:**
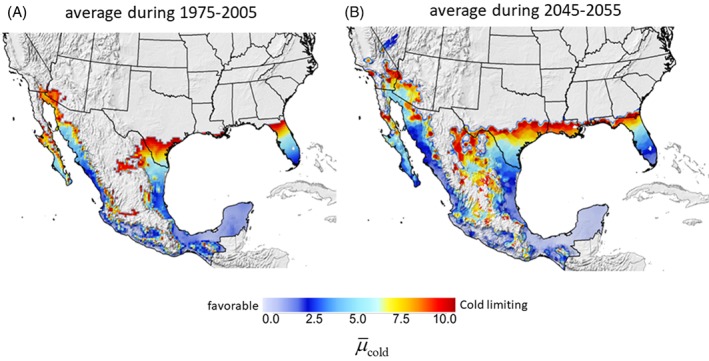
Comparison of areas of screwworm endemicity in North America based on µ_cold_ computed from daily weather data from a high resolution and bias‐corrected climate scenario (Thrasher *et al*., 2012) for (A) the historical period 1975–2005 and (B) the future period 2045–2055. High values of µ_cold_ indicate low favourability, whereas unshaded grey areas are unfavourable (i.e. µ_cold_ > 10). [Colour figure can be viewed at http://wileyonlinelibrary.com].

Cold temperatures limit the potential northward endemic range of the fly, although an increase in average temperatures by year 2050 of 2 °C (or more), as posited by the Intergovernmental Panel on Climate Change (IPCC, Intergovernmental Panel on Climate Change, 2014), would increase its potential endemic range in the U.S.A. However, projections of climate change effects using climate model data are fraught with difficulty because of the complexity of modelling NAMS that make any sound prediction of future weather (Gutzler *et al*., 2005) and fly dynamics problematic. Several regional climate models have been developed (see supplemental materials, File S1). Using high resolution, bias‐corrected NASA climate model data for 2045–2075 vs. 1975–2005 (Thrasher *et al*., 2012; NASA, 2015), the model predicts important increases in the prospective endemic range of screwworm northward into the southern U.S.A. and at higher elevations in Mexico (Fig. 8A vs. B). Under climate change, a wide spread reinvasion would greatly challenge low efficacy SIT intervention, as occurred during the large 1972 outbreak, and as suggested by the predicted outbreaks during 1982–2010 (Fig. 5). Furthermore, climate warming would increase cattle tick populations (and wounds) (Pérez de León *et al*., 2012) and their geographical range, and this would exacerbate the outbreak potential for screwworm. On the positive side, considerable progress has been made with respect to improving SIT eradication technologies (Scott *et al*., 2017), although these advances need to be augmented by an improved forecasting system of screwworm dynamics on fine time and spatial scale across a large geographical region. The development of this system requires that the weather driven biology of the fly (and of cattle tick) be better documented (Gutierrez & Ponti, 2014) than has occurred previously.

## Supporting information


**File S1.** Deconstructing the eradication of new world screwworm in North America.
