# Supplementary material for: Deconstructing the eradication of new world screwworm in North America: retrospective analysis and climate warming effects
Source: Med Vet Entomol. 2019 Feb 13;33(2):282–95. doi: 10.1111/mve.12362 (PMC6849717; doi:10.1111/mve.12362)
Supplement: Supplementary file 1 — File S1. Deconstructing the eradication of new world screwworm in North America. [file MVE-33-282-s001.docx]

**Supplemental materials:**

**Deconstructing the eradication of new world screwworm in North America**

Andrew Paul Gutierrez^1, 2^, Luigi Ponti^1, 3^, Paola A. Arias^4^

^1^Center for the Analysis of Sustainable Agricultural Systems (CASAS Global), 37 Arlington Ave., Kensington, CA, USA 94707-1035

^2^Professor Emeritus, Division of Ecosystem Science, College of Natural Resources, University of California, Berkeley, CA, USA 94720-3114

^3^Agenzia nazionale per le nuove tecnologie, l’energia e lo sviluppo economico sostenibile (ENEA), Centro Ricerche Casaccia, Via Anguillarese 301, 00123 Roma, Italy

^4^Grupo de Ingeniería y Gestión Ambiental (GIGA), Escuela Ambiental, Facultad de Ingeniería, Universidad de Antioquia, Calle 70 No. 52-21, Medellín, Colombia

**Table of contents**

- Myiasis records during the 1962 – 1982 SIT eradication period.
- Weather data - weather data for McAllen, Texas.
- Figure SM1. The ratio of sterile insects released to total cases of myiasis.
- Figures SM2a. Distribution and abundance of Screwworm infestations during the 1962 & 1972, and total cases of myiasis in the USA and Mexico during 1972.
- Figures SM3. Distribution and abundance of Screwworm infestations during the 1962-1980.
- Figures SM4. Distribution and abundance of cattle tick outbreaks in Texas during the 1960-2010 period, and the outbreaks of screwworm during the 1962-1980 period.
- Figures SM5. Eradication dynamics at Tuxtla-Gutierrez during 1990.
- Figure SM 6. The effects of observed weather at Veracruz, Mexico on screwworm oviposition (egg batches/week), and the frequency distribution of egg batches per sentinel pens**.**
- Climate model weather data for assessing climate change effects on screwworm in North America.

**Myiasis records during the 1962 – 1982 SIT eradication period**

The USDA/ARS SIT myiasis records were extracted from photocopy records provided Dr. E.S. Krafsur. Tabulated county level yearly data for screwworm myiasis for the 254 TX counties on request as an EXCEL file from http//CasasGlobal.org at [Casas.Kensington@gmail.com](mailto:Casas.Kensington@gmail.com). Weekly data for 1962 and monthly for 1972 are available for TX. Yearly county data for AR, AZ, CA, Co, Ok, NM are available for 1976-1979.

**Weather data**

Weather data for 1216 locations across the continental U.S.A. and Mexico for the period 1 January 1983 to 31 December 2003 were obtained from: the Global Surface Summary of Daily Weather (GSOD) from the National Climatic Data Center (NCDC) (http://www.ncdc.noaa.gov); Helio-Clim solar radiation data from SoDa (<http://www.soda-is.com/>); E-OBS gridded observed temperature and precipitation data from the ENSEMBLES (<http://ensembles-eu.metoffice.com>) and ECA&D (http://eca.knmi.nl) projects; Gauge-Based Analysis of Global Daily Precipitation data from the Climate Prediction Center (CPC) (http://ftp.cpc.ncep.noaa.gov ); surface meteorology data from the NASA Langley Research Center POWER Project (http://power.larc.nasa.gov) funded by the NASA Earth Science Directorate Applied Science Program; and Daymet data (http://daymet.ornl.gov/).

**Weather data for McAllen, Texas**

Weather data from McAllen (primary station; GHCN-Daily identification code USC00415701) were used as the primary reference station during the 75-year period of 1 January 1942 to 31 June 2017. Missing data for maximum temperature (T_max_, T_min_) and precipitation was 6% of the total. Hence, we used McAllen Miller international airport (USW00012959) less than 1 km away as the backup station but it had 27% missing *T_max_*, *T_min_* and precipitation data during the same time period. After filling missing data in USC00415701 from USW00012959, 2% of T_max_, Tmin and precipitation missing data remained, and these were filled using linear interpolation using the arithmetic mean to replace missing data if and only if valid data were present on the preceding and on the following day relative to the missing value being considered. This procedure left only 10 missing values for precipitation. The closest weather station was more than 28 km away from the primary McAllen station, and hence linear interpolation using the arithmetic mean of the preceding and following 5 days relative at the primary McAllen station were substituted. The few missing precipitation values were embedded series of zero to very low precipitation.

**Figure SM1. The ratio of sterile insects released to total cases of myiasis (log_10_ scale) in Texas.**

**Figures SM2. Annual distribution and abundance of screwworm infestations.**

**Fig. SM2a -- In Texas during 1962.**

GIS maps below show the changes in the observed distribution and abundance of screwworm myiasis cases in Texas with time during the 1962 outbreak. The weekly maps for the first thirteen weeks of data (log scale) are in the first set of figures and, show the beginning outbreak in south Texas on week 7 (from Gutierrez and Ponti 2014 supplemental materials).


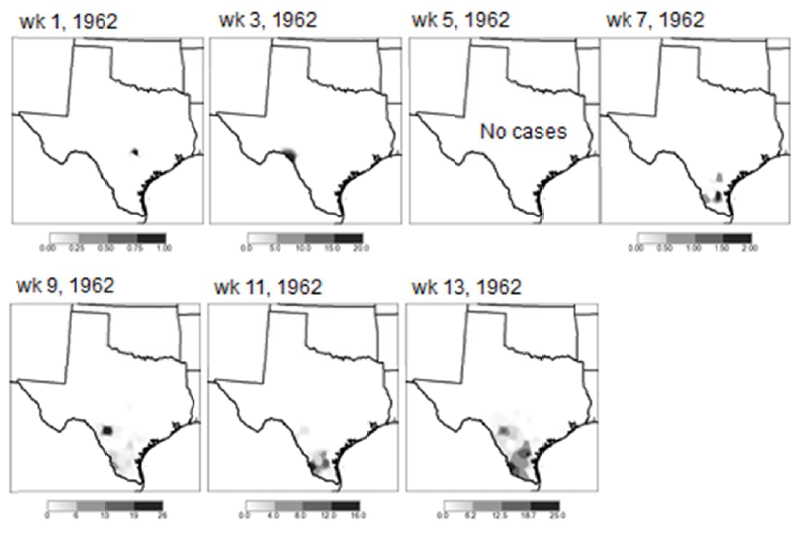


The development of the 1962 outbreak is shown below at 13-week intervals as log_10_(N+1) cases of myiasis. An initial low incidence occurred during January in south Texas along the Rio Grande Valley (see above) and expanded and increased northward during the summer, but declined during the autumn- fall period (weeks 40-52).


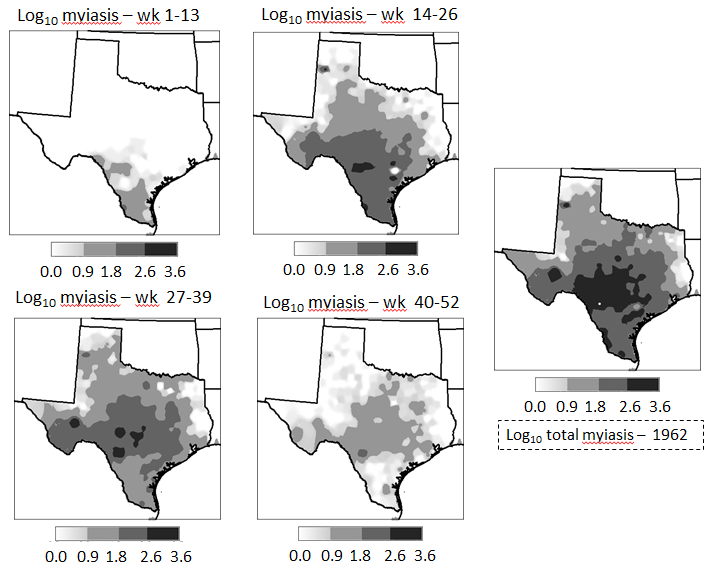


**Fig. SM2B - Total cases of screwworm myiasis in the USA and Mexico during 1972 (below).**


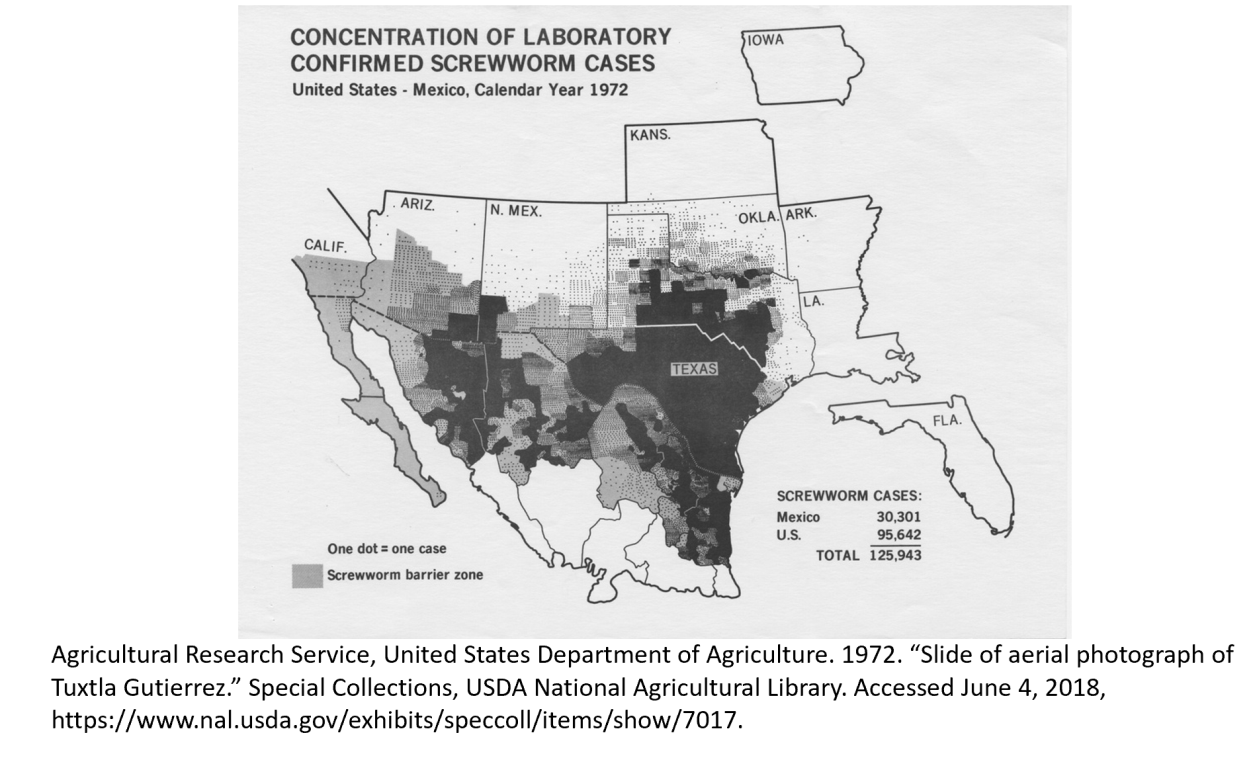


**Figures SM3. Annual distribution and abundance of screwworm myiasis in Texas during 1962-1980.**

The annual observed log_10_ total (N+1) cases of myiasis during 1962 to 1980 are shown below. The black arrow in the 1962 sub figure is the location of McAllen, TX.


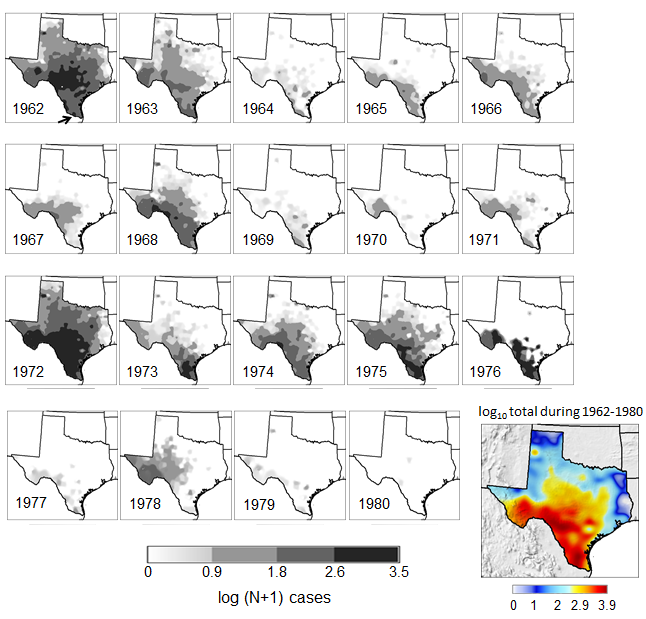


**Figures SM4. Distribution and abundance of cattle tick events (outbreaks) in Texas during the 1960-2010 (A, B) (17).** Note that the patterns for screwworm myiasis (albeit on a higher scale, red fill in B) during the 1962-1979 eradication period are similar to those for tick events.


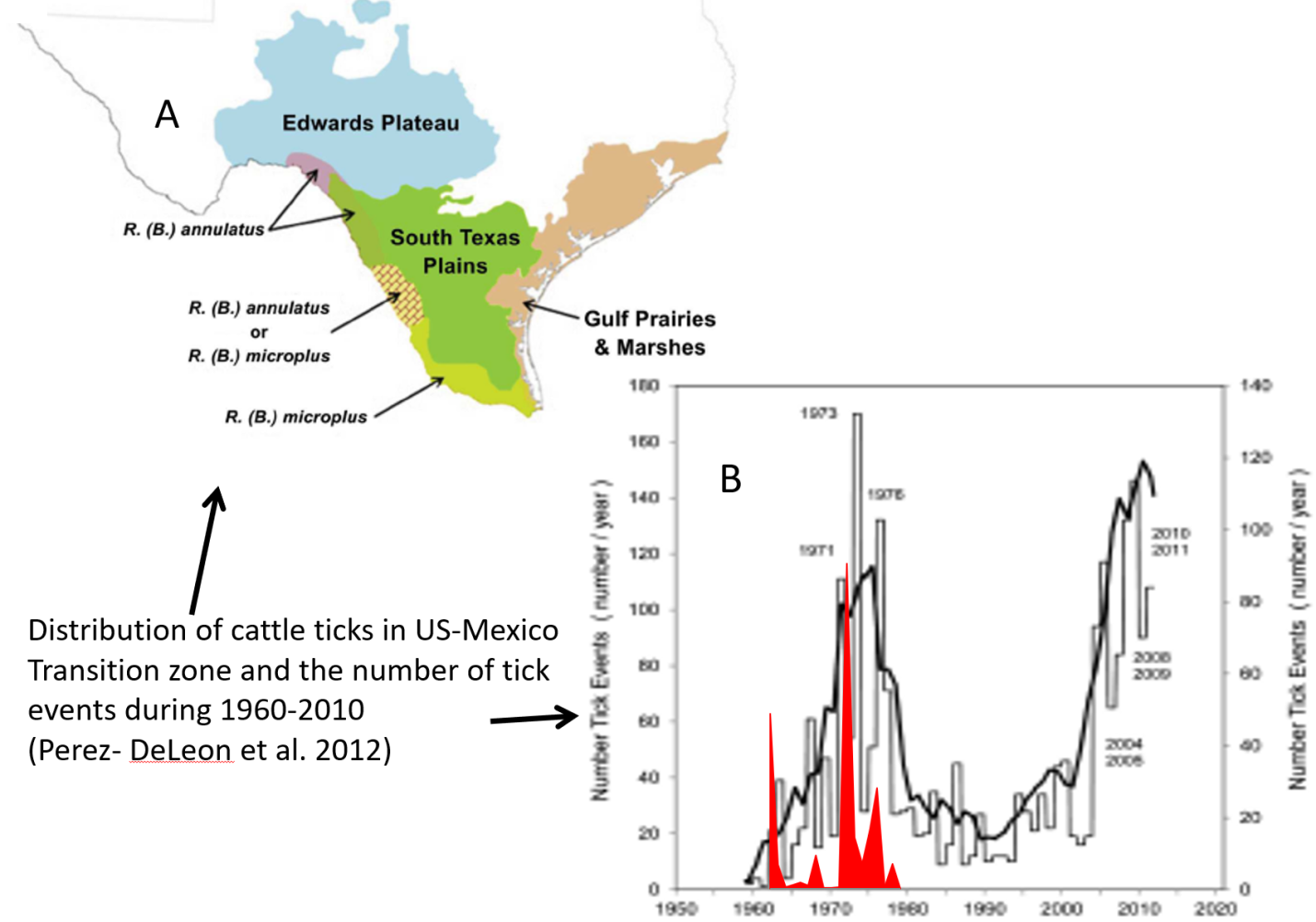


**Figures SM5. Simulated eradication dynamics at McAllen TX and Tuxtla-Gutierrez MX during 1990.**

Using the average of daily adult fly numbers predicted by the simulation as the initial condition (i.e. 6.7 at McAllen TX and 19.7 at Tuxtla-Gutierrez), the required bi-weekly release rates of sterile insects required for eradication within the first season are 15 and 32, respectively. The simulations for McAllen are without SIT (A) and with SIT releases (B), while the simulation for Tuxtla-Gutierrez is only with SIT (C). The first releases were made on 15 January and bi-weekly thereafter. In the model, eradication occurred when the number of all wild fly life stages goes to zero. In the model, immature life stages of the fly serve as refuges from SIT eradication efforts. Note that the time scale for the fly is in physiological time units while the releases are in chronological time explaining the early season overlap of released sterile flies at McAllen (B). In contrast to Tuxtla-Gutierrez (C), the influence of early season cool weather at McAllen on both the non-SIT and SIT eradication dynamics delays population growth (A) and eradication (B).


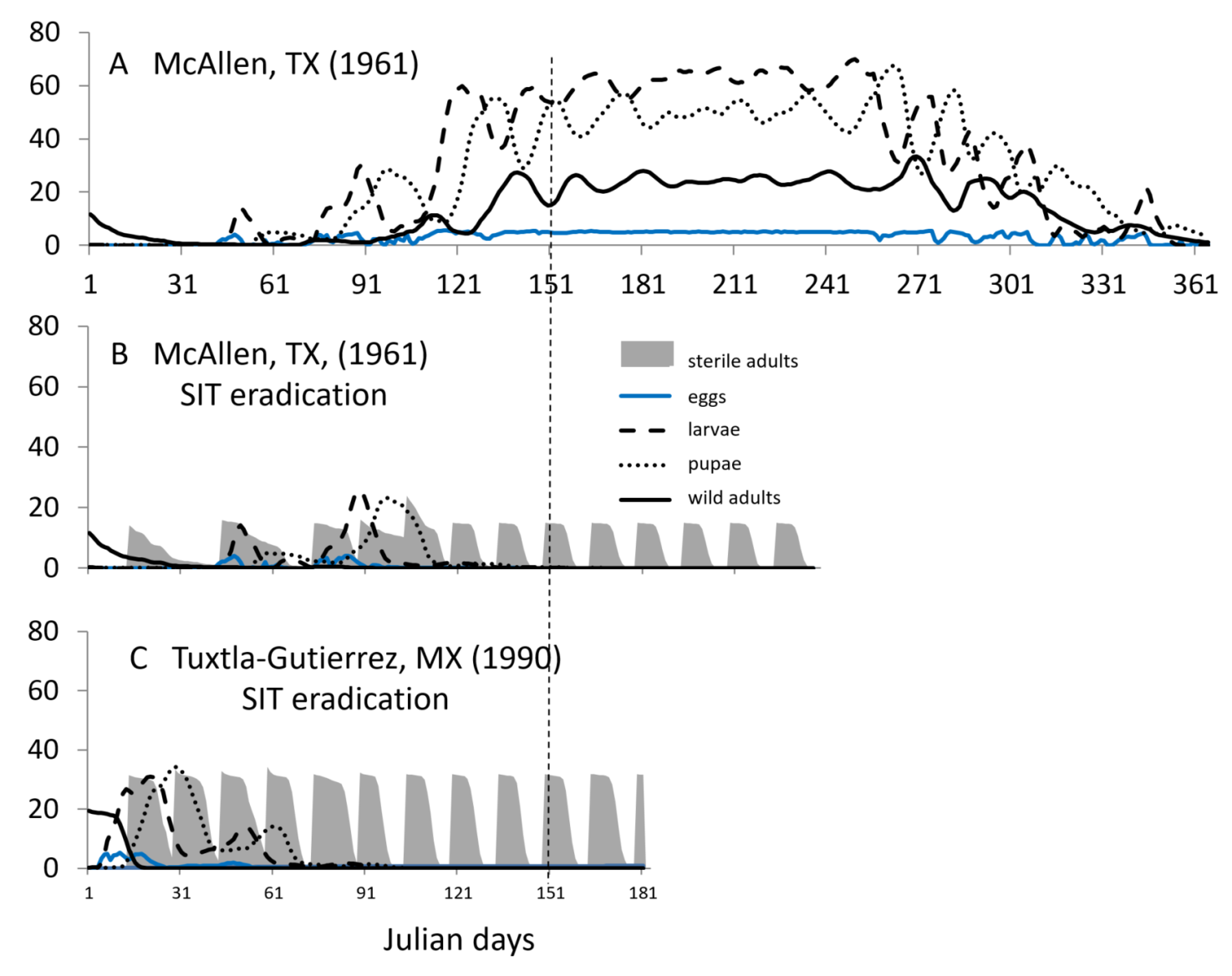


**Figure SM 6. Reanalysis of the effects of observed weekly temperature and rainfall at Poza Rica, Veracruz, Mexico on (A, B) screwworm oviposition (egg batches/week), (C) daily adult mortality rate (μ_adult_), and (D) the frequency distribution of egg batches per sentinel pen (summarized from Krafsur *et. al.* 1979).**

Field data on egg masses verify aspects of the biology in the PBDM (see text). Specifically, egg mass production increases with temperature above 14.5C, there is no direct correlation of oviposition to weekly rainfall (B) though some correlations were found with time lags, (C) the adult mortality rate (*μ_adult_*) increases 8-10fold during the cooler winter period, and (D) oviposition of egg mass on penned animals appears to be random.


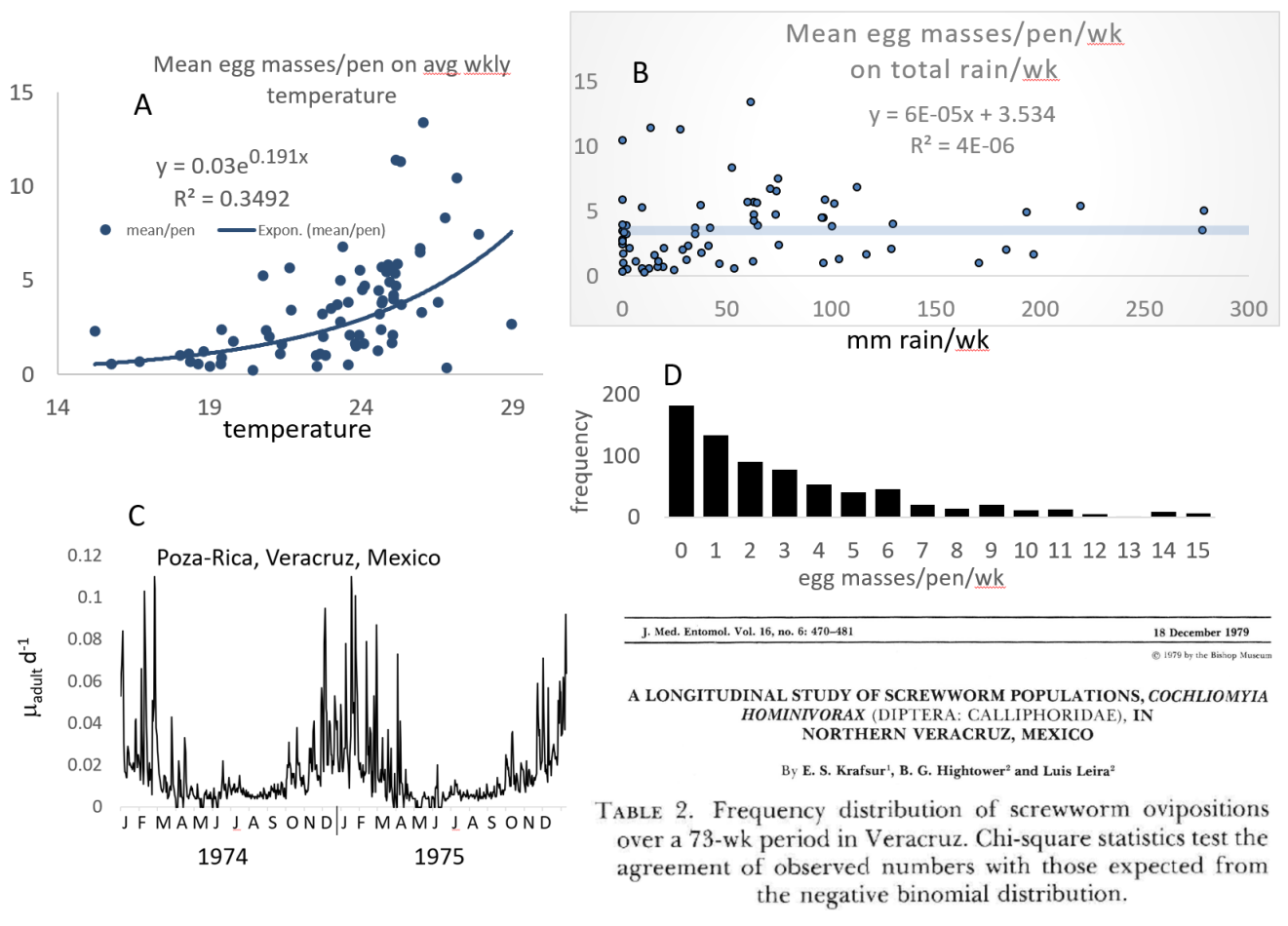


# Climate model weather data for assessing climate change effects on screwworm in North America

Many climate models are challenged by the complexity of the North American monsoon system (NAM) system (Gutzler *et. al.* 2005). Bukovsky *et. al.* (2013) reviewed the various climate models of the North American Regional Climate Change Assessment Program (NARCCAP) (see Mearns *et al.*, 2009; <http://www.narccap.ucar.edu/about/model-changes.html>). Of these, Bukovsky *et. al.* (2015) surmised that the HRM3+HADCM3 regional model was the most credible simulation for NAM projecting small but significant decreases in mean precipitation during the core of the NAM season across the Southwestern US, small increases in the number of dry days regionally, and an increase in the frequency of the heaviest precipitation events with a decrease in the frequency of precipitation of lesser intensities. It is important to note that while the more credible simulations generally produced a smaller signal for a decrease in mean NAM precipitation amount by midcentury, this would not necessarily preclude drying in the region, as temperatures are also projected to rise, and soil moisture evaporation would increase. Bukovsky *et. al.* (2015) also caution that it would be difficult to assess whether any of the NARCCAP simulations should be used for any purpose over North America aside from general research on model results. However, HRM3 shows the greatest overall temperature bias when compared to the other NARCCAP regional models (Bukovsky 2012, Mearns *et. al.* 2012). Further, Mearns *et. al.* (2015) point out that NARCCAP model simulations are mainly of value to impacts researchers when used to explore the uncertainty in climate change impacts as related to the spatial scale of climate projections. When NARCCAP simulations are used in raw form (i.e., without bias correction) to drive impact assessments, the results will in general not resemble those obtained when using actual observed climate as input, and this makes bias correction a de facto requirement (Mearns *et. al.* 2015). For example, HRM3 exhibits the largest warm biases with magnitudes exceeding 8°C over much of North America (Loikith *et. al.* 2014). Many NARCCAP regional climate models including HRM3 also show bias related to the tails of model-simulated temperature distribution, and this will impact the accuracy with which models simulate extremes that is harder to account for via bias correction (Loikith *et. al.* 2014).

The NAM is a complex circulation system made up of well-defined large-scale to mesoscale seasonal circulation features (Mearns *et. al.* 2016). It is responsible for more than half of the annual precipitation that falls in northwestern Mexico and southern Arizona, and significant quantities in the rest of the southwest United States as well. There is a clear difference of opinion on whether projections from one regional climate model (RCM) nested within one global climate model (GCM) would provide credible information—even when no policy planning or decisions would be made. However, the results of the expert judgment (surveys) contrast somewhat with the ranking of the RCMs produced using a set of generally applicable quality metrics that were initially developed for weighting the European ENSEMBLES set of RCM simulations (see Mearns *et. al.* 2016).

Luong *et. al.* (2017) suggests that existing global and regional climate change models do not represent the NAM system well in either seasonal forecasts or climate projections, which is why in their paper they used historical observed weather instead to study NAM. They looked for extreme rainfall events during 1950-1970 as compared with 1991-2010. Average precipitation was about the same, but 1991-2010 had more storms with very heavy rain.

We attempted to use the HRM3–HADCM3 data set from NARCCAP simulations (<http://www.narccap.ucar.edu>). NARCCAP simulations are forced with the SRES A2 emissions scenario for the 21st century. RCMs were nested within the Global Climate Models (AOGCMs) for the current period 1971-2000 and for the future period 2041-2070 and were run at 50km spatial resolution. Results for seasonal average climate change in temperature and precipitation for the driving HADCM3 GCM and for the HRM3 RCM driven with HADCM3 boundary conditions are found at <http://www.narccap.ucar.edu/results/seas-delta-maps/hrm3-hadcm3-results.html> (see Mearns *et al.*, 2014).

**References**

Bukovsky MS. 2012. Temperature trends in the NARCCAP regional climate models. *Journal of Climate* 25:3985–3991. DOI: 10.1175/JCLI-D-11-00588.1

Bukovsky MS., Gochis DJ., Mearns LO. 2013. Towards assessing NARCCAP regional climate model credibility for the North American monsoon: current climate simulations. *Journal of Climate* 26:8802–8826. DOI: 10.1175/JCLI-D-12-00538.1.

Bukovsky MS., Carrillo CM., Gochis DJ., Hammerling DM., McCrary RR., Mearns LO. 2015. Toward assessing NARCCAP regional climate model credibility for the North American monsoon: future climate simulations. *Journal of Climate* 28:6707–6728. DOI: 10.1175/JCLI-D-14-00695.1.

Gutzler DS., Kim H-K., Higgins RW., Juang H-MH., Kanamitsu M., Mitchell K., Mo K., Pegion P., Ritchie E., Schemm J-K., Schubert S., Song Y., Yang R. 2005. The North American monsoon model assessment project: integrating numerical modeling into a field-based process study. *Bulletin of the American Meteorological Society* 86:1423–1429. DOI: 10.1175/BAMS-86-10-1423.

Loikith PC., Waliser DE., Lee H., Kim J., Neelin JD., Lintner BR., McGinnis S., Mattmann CA., Mearns LO. 2014. Surface temperature probability distributions in the NARCCAP hindcast experiment: evaluation methodology, metrics, and results. *Journal of Climate* 28:978–997. DOI: 10.1175/JCLI-D-13-00457.1

Luong TM., Castro CL., Chang H-I., Lahmers T., Adams DK., Ochoa-Moya CA. 2017. The more extreme nature of North American monsoon precipitation in the southwestern U.S. as revealed by a historical climatology of simulated severe weather events. *Journal of Applied Meteorology and Climatology*:DOI: 10.1175/JAMC-D-16-0358.1. DOI: 10.1175/JAMC-D-16-0358.1.

Mearns LO., Gutowski W., Jones R., Leung R., McGinnis S., Nunes A., Qian Y. 2009. A regional climate change assessment program for North America. *Eos, Transactions American Geophysical Union* 90:311–311. DOI: 10.1029/2009EO360002.

Mearns LO., Arritt R., Biner S., Bukovsky MS., McGinnis S., Sain S., Caya D., Correia J., Flory D., Gutowski W., Takle ES., Jones R., Leung R., Moufouma-Okia W., McDaniel L., Nunes AMB., Qian Y., Roads J., Sloan L., Snyder M. 2012. The North American regional climate change assessment program: overview of phase I results. Bulletin of the American Meteorological Society 93:1337–1362. DOI: 10.1175/BAMS-D-11-00223.1

Mearns L., McGinnis S., Arritt R., Biner S., Duffy P., Gutowski W., Held I., Jones R., Leung R., Nunes A., Snyder M., Caya D., Correia J., Flory D., Herzmann D., Laprise R., Moufouma-Okia W., Takle G., Teng H., Thompson J., Tucker S., Wyman B., Anitha A., Buja L., Macintosh C., McDaniel L., O’Brien T., Qian Y., Sloan L., Strand G., Zoellick C. 2007, updated 2014. North American Regional Climate Change Assessment Program dataset. DOI: 10.5065/D6RN35ST. Accessed 2017-08-17.

Mearns LO., Lettenmaier DP., McGinnis S. 2015. Uses of results of regional climate model experiments for impacts and adaptation studies: the example of NARCCAP. *Current Climate Change Reports* 1:1–9. DOI: 10.1007/s40641-015-0004-8.

Mearns LO., Bukovsky MS., Schweizer VJ. 2016. Potential value of expert elicitation for determining differential credibility of regional climate change simulations: an exercise with the NARCCAP co-pis for the southwest monsoon region of North America. *Bulletin of the American Meteorological Society* 98:29–35. DOI: 10.1175/BAMS-D-15-00019.1.
